# Memory and communication efficient algorithm for decentralized counting of nodes in networks

**DOI:** 10.1371/journal.pone.0259736

**Published:** 2021-11-22

**Authors:** Arindam Saha, James A. R. Marshall, Andreagiovanni Reina

**Affiliations:** 1 Department of Computer Science, University of Sheffield, Sheffield, United Kingdom; 2 IRIDIA, Université Libre de Bruxelles, Brussels, Belgium; University Campus Bio-Medico of Rome, ITALY

## Abstract

Node counting on a graph is subject to some fundamental theoretical limitations, yet a solution to such problems is necessary in many applications of graph theory to real-world systems, such as collective robotics and distributed sensor networks. Thus several stochastic and naïve deterministic algorithms for distributed graph size estimation or calculation have been provided. Here we present a deterministic and distributed algorithm that allows every node of a connected graph to determine the graph size in finite time, if an upper bound on the graph size is provided. The algorithm consists in the iterative aggregation of information in local hubs which then broadcast it throughout the whole graph. The proposed node-counting algorithm is on average more efficient in terms of node memory and communication cost than its previous deterministic counterpart for node counting, and appears comparable or more efficient in terms of average-case time complexity. As well as node counting, the algorithm is more broadly applicable to problems such as summation over graphs, quorum sensing, and spontaneous hierarchy creation.

## Introduction

All decentralized systems share the common aspect of being comprised of a network of units (which can be considered as graph nodes) that rely on local and partial information which they can gather from the subset of devices in their communication range (communication links can be represented as graph edges). An open challenge is to allow the units of these large-scale decentralized systems to estimate properties of the entire group.

A fundamental property that is crucial for the design and the efficient functioning of several systems is the system size, that is, the number of units in the system. Computing the exact network size in finite time with a decentralized algorithm with finite complexity is proved to be impossible [1]. Previously proposed solutions are therefore stochastic algorithms that only give an approximation of the system size, providing the possible advantages of robustness and speed. Deterministic algorithms provide the exact solution in a finite time, however, they may rely on stringent assumptions on the communication network topology. An overview of the existing algorithms is provided in Section *State of the art*. We propose, in Section *The aggregate-and-broadcast algorithm*, a new decentralized deterministic algorithm, the *aggregate-and-broadcast* (AnB) algorithm, that iteratively aggregates the node counts into a small number of local hubs which finally broadcast the count throughout the whole network. The AnB algorithm allows the nodes to compute the exact network size in a finite time when an upper bound is provided. In other words, the network size computed by the AnB algorithm is exact up to a limit that is bounded by the algorithm’s execution time, as proved in the S1 File. The algorithm relies on the only two assumptions of a connected network and uniquely identifiable units (i.e. unique id), and requires minimal computation and communication capabilities of the units. The algorithm performance is analyzed and when possible compared with previous algorithms in terms of time, communication, and memory costs (see Section *Analysis of the algorithm*). The results indicate that the AnB algorithm is scalable, efficient, and accurate, with better performance than the existing algorithms in terms of smaller memory and communication costs. Therefore, as discussed in the *Conclusion*, the AnB algorithm can be beneficial for systems with constrained memory and communication, and has the potential to be employed in numerous application cases and impact a large variety of decentralized systems.

## The problem statement

Consider a connected network G=(V,E), where V={1,…,N} is the set of nodes in the network and E⊆V×V is the set of the edges of the network. The edges describe undirected and unweighted communication links between nodes, i.e. (*u*, *v*) ⇔ (*v*, *u*) ∈ *E*. Each node can only communicate at synchronous timesteps with its neighbors, where the set of neighbors of the generic node *v* is defined as $N_{i}=\left\{u\in V|\left(v,u\right)\in E\right\}$. We assume G to be time-invariant. Each node is characterized by a unique identifier (id). Each node knows an upper bound *N*_max_ of the network size, such that *N*_max_ ≥ *N*. In this paper, we propose an algorithm to be executed by every node of the network to allow them to compute the network size *N* in a finite number of iterations *t*_max_ ≤ 4*N*_max_ + 1 (and therefore, a finite amount of time). Note that knowledge about *N*_max_ is only necessary in order to bound the number of iteration steps required for the execution of the algorithm to *t*_max_. This is required due to the results reported by Hendrickx et al. [1] who have proved that it would be otherwise impossible for a finite complexity algorithm to correctly count the number of nodes (see discussion in Sec. Stopping criteria).

## State of the art

Most of the algorithms proposed to estimate the size of the network rely on stochastic methods. The most common approach relies on executing variations of random walks on the network [2–5]. In particular, Ganesh et al. [2] used continuous time random walks to obtain a target number of redundant node samples. The time required to obtain such a sample was then used to estimate the network size. In a different study, Gjoka et al. [3] compared various weighted random walk techniques. The study identified efficient methods to identify various macroscopic properties of the network by simulating weighted random walks on the network (e.g. Metropolis-Hastings Random Walk and Re-Weighted Random Walk). Similarly, Katzir et al. [4] proposed a method based on simulating multiple simultaneous random walks in order to estimate the size of the network. Building upon this work, Musco et al. [5] proposed an algorithm where multiple nodes execute random walks and compute the network size based on the degrees of the nodes encountered. Notable stochastic algorithms which do not involve random walks rely on either average consensus [6] or on order statistics consensus [7–10].

One of the shortcomings of stochastic algorithms is that their run-times depend on the desired accuracy of the results. Therefore, for applications where the size of the network is required to a high degree of accuracy, stochastic algorithms might take a long time to converge. For instance, the number of dynamical attractors in Boolean networks and their periodicities depend on whether the network size is even or odd, prime or composite [11]. Since dynamics on such networks are crucial in studying social networks, neural networks and gene and protein interaction networks [12–16], accurate knowledge of the network size is crucial. In such scenarios, deterministic algorithms to estimate the network size are better suited.

To the best of our knowledge, the number of deterministic algorithms for decentralized network node counting is very limited. One of the most trivial algorithms is the *All-2-All* method, as alluded to in Ref. [17]. It consists in having each node broadcasting a unique id together with all ids that it has already received so far. This simple algorithm is the most efficient algorithm we are aware of for deterministic network node counting on general network topologies. Other algorithms for node counting have been proposed for networks with specific topologies. For example, an algorithm inspired by the Breadth-First-Search (BFS) algorithm can be used on a tree network. In 2003, Bawa et al. [18] generalized such an algorithm so that it could be implemented on a network with a general topology. In their paper, the authors propose three different algorithms which may be used for computing various aggregates across the network. While the proposed algorithms are efficient, they investigated a different problem. They focus on the situations when the network size or the other aggregate quantities are sought by a single node of the network. When every node requires the size information, repeating the algorithm of [18] on every node becomes less efficient than the All-2-All method, as described in Sec. Analysis of the algorithm. Notably, numerous algorithms have been proposed to create a spanning tree on a general network. However, they are constrained in a crucial aspect as underlined in the next section.

## Significance of the work

In Ref. [18], the authors propose algorithms to create a spanning tree on the given network. Once a tree is constructed, any information can be aggregated in the root by following the edges of the tree. Due to its important applications, numerous other algorithms [19–30] have also been proposed to construct a spanning tree on a connected network. All these algorithms, in order to build a spanning tree, require that one node of the network assumes the role of the root of the tree. However, selecting one node to assume such a role through a decentralized algorithm running on a sparse network is a difficult problem on its own. In fact, the network nodes would need to invest resources (time and computation) to reach a consensus on a single root node and avoid duplicates.

In this paper, we present an algorithm to create a ‘tree-like’ network to span a general connected network without assuming any particular node as a root. Instead of generating a tree from the root, our algorithm removes edges consecutively based on the local neighborhood of each node. This results in the emergence of possibly multiple ‘root-like’ nodes (which we call ‘residue’ nodes). Any information which was initially distributed among all nodes of the network can therefore be concentrated in these residue nodes. Thereafter, the information can be broadcast throughout the network.

In addition to relaxing the restriction of a selected root, the AnB algorithm performs better than the other known algorithms in terms of communication and memory costs than the existing algorithms. In fact, typically, the AnB algorithm, by creating multiple root-like nodes, decentralizes the computation to different parts of the network and thus nodes use on average less memory and send fewer messages. Our empirical analysis shows that the time costs of the algorithm depend crucially on the network topology: the proposed algorithm performs better than the previous algorithms for large random geometric networks but worse than them for other types of network topology. Hence, the AnB algorithm may prove to be useful in networks where the required memory per node is the major limiting factor or the limited communication between nodes is desirable.

Additionally, the proposed algorithm can be used to perform other collective tasks where aggregation of information is required but a distinguished root node cannot be identified (see the Conclusion).

## The aggregate-and-broadcast algorithm

We propose the aggregate-and-broadcast (AnB) algorithm, a deterministic algorithm for the simultaneous and decentralized determination of the size *N* of a finite connected network by all its nodes. We assume that each node of the network has a unique id, can communicate only with its immediate neighbors, and knows *N*_max_, the upper bound of the network size. Other than that, we make no prior assumptions about the topology of the network nor prior knowledge of the node. The underlying idea of the AnB algorithm is inspired by the standard node-counting method on a tree by its root. In a tree, the counts of the leaves are assimilated by their respective parents and then the leaves are iteratively pruned. Applying such an algorithm on a graph with a general topology poses a challenge since a strict hierarchy does not exist among the nodes. To overcome this problem, we add a step in each iteration where, based on the degree of its neighbors, each node determines its local hierarchy which, in turn, determines whether it should be pruned or not.

In the next subsections, we describe the proposed AnB algorithm in detail. We start with an overview of the entire algorithm in the next subsection. In subsections Pre-iteration steps and Iteration steps, we describe the pre-iteration steps (which include variable initialization) and the iteration steps of the algorithm respectively. Finally, in subsection Remarks on the AnB algorithm we compare the AnB algorithm to the standard node counting algorithm in trees and make some further remarks about the proposed algorithm. The correctness of the AnB algorithm is proved in Sec. Theorems and Proofs of the S1 File.

### An overview of the AnB algorithm

Prior to the iterative steps, the nodes of the network are initialized as follows. The behavior of a node with id *i* at any particular instant is determined by its state *s*_*i*_ which can take one of four values during the course of the algorithm: ‘active’ (*A*), ‘leaf’ (*L*), ‘residue’ (*R*), or ‘inactive’ (*I*). The state of each node is initialized to *s*_*i*_ = *A*. Each node also starts with a local node counter *c*_*i*_ = 1. Since, at the beginning of the algorithm, each node is aware only of its own existence, the counter is initialized to 1. As the algorithm progresses, the node gathers information about the changing state of nodes (equivalent to the nodes getting ‘pruned’) from its neighbors and updates the value in *c*_*i*_. Additionally, each node also has the following other internal variables: the set of its neighbors $N_{i}$, its effective neighborhood $E_{i}$, effective degree *e*_*i*_, the set of residues $R_{i}$ and final node count *n*_*i*_. Among these, the first three variables are initialized to be empty sets $N_{i}=E_{i}=R_{i}=\emptyset$, and the effective degree and final count variable are initialized as *e*_*i*_ = *n*_*i*_ = 0. Note, that it is assumed that the nodes of the network are synchronised and have a common sense of time. In other words, the nodes are aware of the beginning and end of each iteration step of the algorithm. Therefore, implementation of the AnB algorithm on a distributed system, e.g., a robot swarm or sensor network, will also need a mechanism to guarantee that synchronization is achieved and maintained.

From the perspective of a node, the AnB algorithm is divided into two phases: ‘pre-reduction’ and ‘post-reduction’. A node is said to be in pre-reduction phase when its state is either *s*_*i*_ = *A* or *s*_*i*_ = *L*. As this phase progresses, a node in ‘active’ state updates its local counter *c*_*i*_ by locally accumulating information from ‘leaf’ neighbors getting ‘pruned’ until the node itself changes its state to *s*_*i*_ = *L* and becomes a ‘leaf’ node. Note that, here the term ‘leaf’ is used to denote a node which is about to be ‘pruned’ from the network; and not necessarily a node with only one neighbor. In the next iteration, each leaf node, depending on their effective neighborhood $E_{i}$, again changes its state to either (a) *s*_*i*_ = *I* and gets ‘pruned’, or (b) *s*_*i*_ = *R* and becomes a residue node.

At the end of pre-reduction phase, the nodes of the network are either in residue (*s*_*i*_ = *R*) or inactive (*s*_*i*_ = *I*) states. These states can be considered analogous to the ‘root’ and the ‘pruned leaves’ of a tree network respectively. The residue nodes contain parts of the total count of nodes in the network. This is similar to the root of a tree network which contains the total node count of the entire tree after all the nodes have been pruned. This information is then broadcast across all other nodes and assimilated to give the final node count of the network. To do this, each residue node constructs a ‘broadcast message’ *b*_*i*_, sends it to all its neighbors and changes its state to *s*_*i*_ = *I*. This broadcast message is then relayed by all nodes—irrespective of their state *s*_*i*_—across the network. A node that receives a broadcast message adds the partial count to its final count variable *n*_*i*_, and keeps track of the residue nodes to avoid double counting. Thus, after iteration steps *t*_max_, the variable *n*_*i*_ gives the total count of all nodes in the network. Further details of the algorithm and the the stopping criteria are provided in subsections Iteration steps and Stopping criteria respectively.

**Algorithm 1:** The aggregate-and-broadcast (AnB) algorithm for network node counting



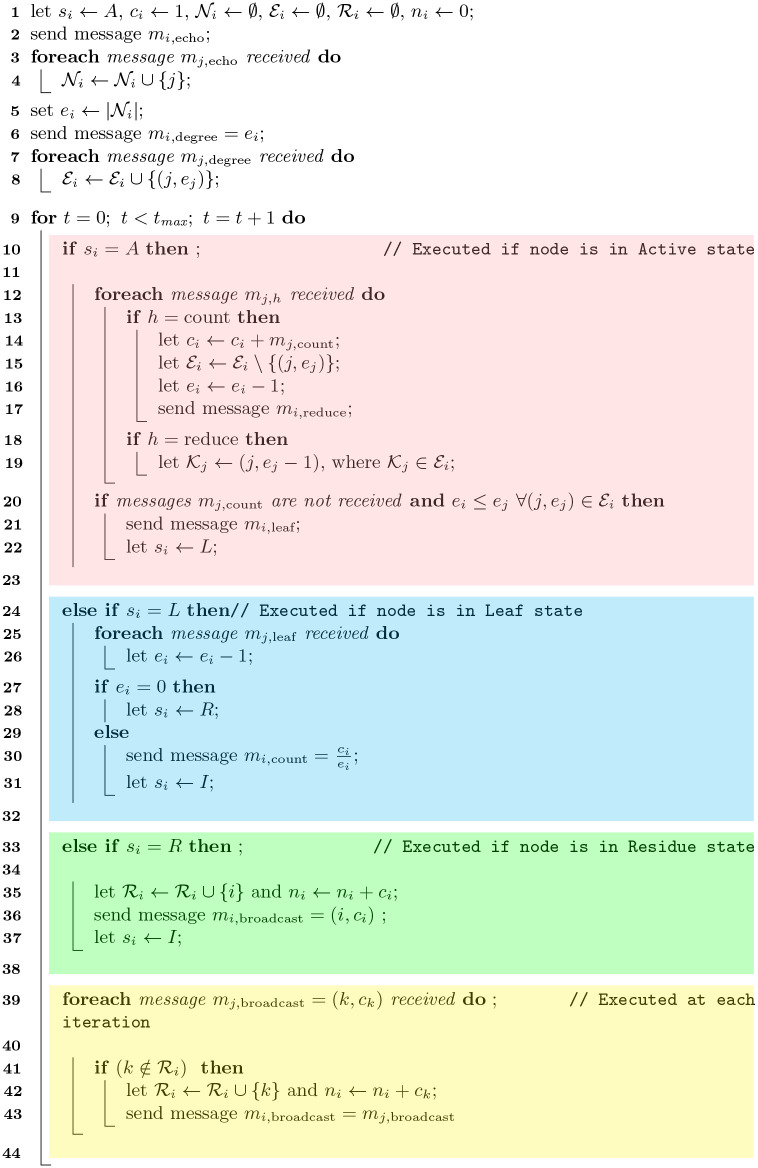



### Pre-iteration steps

We now describe the AnB algorithm in detail. The actions taken by a node *i* in a particular step are determined by its internal variables and the messages it receives from its neighbors, *i.e.* the nodes in $N_{i}$.

Any message sent by a node is denoted as *m*_*i*,*h*_, where *i* is the sender of the message and *h* is the ‘type’ of the message. The ‘type’ of the message determines the action to be taken by the receiver of the message. The various types of messages and their roles are summarized in Table 1. Note that every message is broadcast to the entire neighborhood $N_{i}$ and thus, can be accessed by all nodes in $N_{i}$.

**Table 1:** The different types of messages *m*_*i*,*h*_ used in the AnB algorithm.

*h*	Content	Role of the message
echo	-	Indicates the presence of the sender *i*
degree	*e*_*i*_	Sends the initial effective degree *e*_*i*_ of the sender *i*
leaf	-	Indicates the transition of the sender *i* to leaf state
count	*c*_*i*_	Sends the local count *c*_*i*_ of the sender *i*
reduce	-	Indicates the reduction of effective degree *e*_*i*_ of the sender *i*
broadcast	(*k*, *c*_*k*_)	Sends or relays the broadcast message

After the initialization of all internal variables, each node of the network identifies its neighborhood. To do so, it sends a message *m*_*i*,echo_ indicating its presence to all its neighbors. It then receives similar messages *m*_*j*,echo_ from other nodes. The set of all nodes from which such a message is received is then identified as the neighborhood $N_{i}$ (Line 4).

One of the most crucial internal variables for the node is its effective degree *e*_*i*_ which is the number of its neighbors which are in the active state (*s*_*i*_ = *A*). Since all nodes start in the active state, the initial effective degree of the node is the number of elements in its neighborhood: $e_{i}=|N_{i}|$. In addition to its own effective degree, the node also needs to be aware of the effective degrees of those neighbors which are in active state. The node keeps track of this information in form of its effective neighborhood,

$$\begin{array}{c} E_{i}=\{\left(j,e_{j}\right):j\in N_{i}\;\text{and}\;s_{j}=A\}. \end{array}$$ (1)

Therefore, $E_{i}$ is a set of tuples where the first element of the tuple is the id of an active neighbor of *i* and the second element is the effective degree of the neighbor.

The identification of neighborhood also allows the node to compute its initial effective degree $e_{i}=|N_{i}|$ and to send it to its neighbors as *m*_*i*,degree_. Thereafter, a node *i* receiving a message *m*_*j*,degree_ updates its effective neighborhood $E_{i}$ as described in Line 8.

### Iteration steps

**Fig 1:**
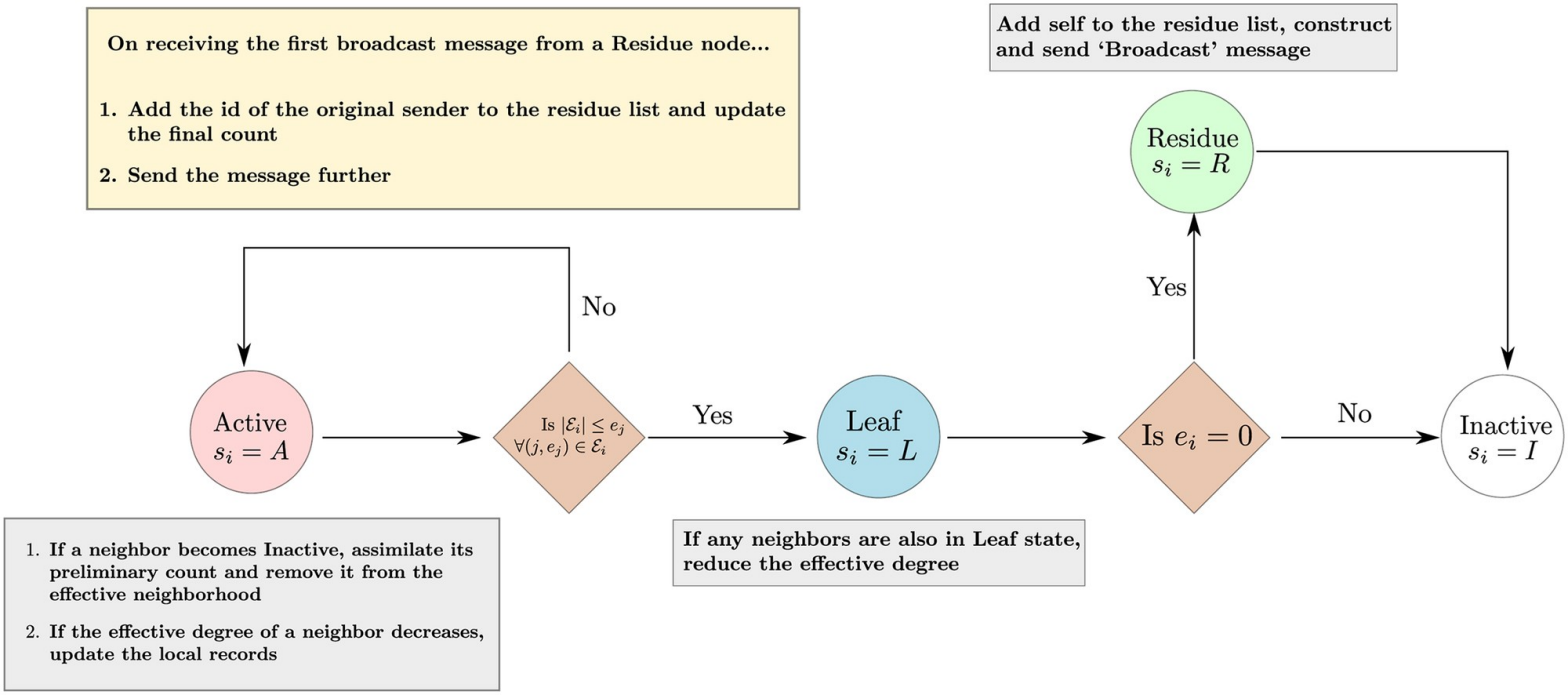
Schematic flowchart depicting the finite state machine of each node of the network executing the AnB algorithm. Note that the colors of the circles correspond to the colors of the section in Algorithm An overview of the AnB algorithm. Also, the steps outlined in the yellow box are carried out by all nodes irrespective of their state.

After the pre-iteration steps, the node *i* enters an iterative phase where its steps are determined by its state *s*_*i*_. The details of these state-dependent steps are illustrated in the finite state machine of Fig 1 and are elaborated as follows.

- **Active nodes:** Each active node *i* with *s*_*i*_ = *A* first detects any change in its neighborhood. This change can be of two types: (a) Either some of its neighbors are transitioning to inactive state (message with *h* = count); or (b) the effective degree of some of its neighbors is being reduced (message with *h* = reduce). Therefore, upon receipt of a message *m*_*j*,count_, the node *i* excludes the sender from its effective neighborhood $E_{i}$, decreases its effective degree *e*_*i*_ by 1 and assimilates the contents of the message in its local count (Line 14),
  $$\begin{array}{c} c_{i}=c_{i}+m_{j,\text{count}}. \end{array}$$ (2)
  Since the effective degree of node *i* is decreased by 1, it sends a message *m*_*i*,reduce_ to its neighbors. For each message of type *h* = 5 received, the node updates the record of the effective degree corresponding to the sender of the message (Line 19).
  After processing the incoming messages, the node *i* checks for the two conditions indicated in Line 20. If both conditions are met, the node sends a message *m*_*i*,leaf_ and changes its state to *s*_*i*_ = *L*; otherwise, the node stays in the active state for the next iteration.
- **Leaf nodes:** The node *i* in state *s*_*i*_ = *L* stays in this state for exactly one iteration and then changes its state to either *s*_*i*_ = *R* or *s*_*i*_ = *I*. First, it processes any incoming message of the type *h* = leaf. The reception of any such message implies that some of its neighbors have transitioned to the leaf state in the same time step, and are therefore no longer in the active state. For each message *m*_*j*,leaf_ received, the effective degree *e*_*i*_ of the node is reduced by one. After processing all incoming messages, the node *i* changes its state; if the effective degree *e*_*i*_ = 0, it change its state to *s*_*i*_ = *R* otherwise, it sends the message
  $$\begin{array}{c} m_{i,\text{count}}=\frac{c_{i}}{e_{i}} \end{array}$$ (3)
  and changes state to *s*_*i*_ = *I* (Lines 27–31).
- **Residue nodes:** Each node *i* in state *s*_*i*_ = *R* updates its residue set $R_{i}$ with its own id *i* and the total node counter *n*_*i*_ adding its local counter *c*_*i*_. It then broadcasts a message *m*_*i*,broadcast_ = (*i*, *c*_*i*_) and changes its state to *s*_*i*_ = *I*.
- **All nodes:** While the previous steps are executed by nodes in a specific state, the following steps are executed by all nodes of the network at each iteration irrespective of their state. Whenever a node *i* receives a message *m*_*j*,broadcast_ = (*k*, *c*_*k*_) from any of its neighbors, it checks if node *k* is in the residue set $R_{i}$. If $k\notin R_{i}$, the node *i* adds *k* to its residue set $R_{i}=R_{i}\cup \left\{k\right\}$, adds the corresponding local count *c*_*k*_ to its final node count *n*_*i*_ = *n*_*i*_ + *c*_*k*_ and finally relays the message forward by sending message *m*_*i*,broadcast_ = *m*_*j*,broadcast_.

**Fig 2:**
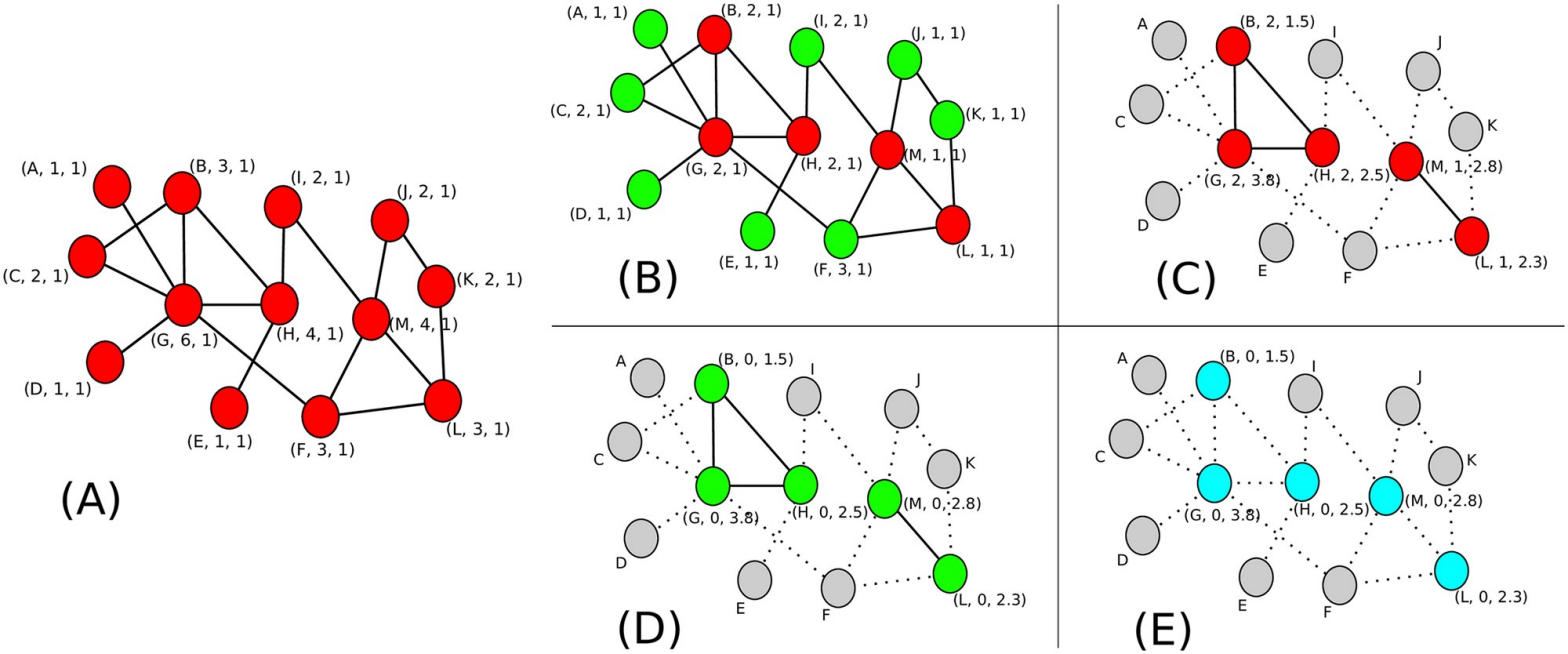
Demonstration of the AnB algorithm on a typical network. Panel (A) shows the initial state of a network of size 13. Panels (B) through (E) show the successive steps in the execution of the aggregate phase of the algorithm. The nodes in Active, Leaf, Residue and Inactive states are shown in red, green, cyan and grey colors respectively. The bracket beside each node shows the id *i* of the node, the number of its active neighbors *e*_*i*_, and its current local count *c*_*i*_, as an ordered tuple. For inactive nodes, only the id of the node is shown because the quantities *e*_*i*_ and *c*_*i*_ are not required in the inactive state. Once the network reaches a state where only residue and inactive nodes are present in the network (as seen in Panel E), the AnB algorithm enters its broadcast phase and the individual local counts of the residue nodes are broadcast throughout the network.

After a sufficient number of iteration steps *t*_max_, all nodes converge to the same final count *n*_*i*_ equal to the network size *N*. A detailed analysis of the convergence time is provided in Sec. Time Cost of the S1 File. An illustration of the working of the aggregate phase of the AnB algorithm is shown in Fig 2.

### Stopping criteria

The AnB algorithm terminates when sufficient iteration steps, *t*_max_, has passed. This *t*_max_ should be sufficiently large so that each broadcast message reaches every node of the network. However, determining an exact value for *t*_max_ is impossible as reported by Hendrickx et al. [1] who have shown that it is impossible for a finite complexity algorithm to correctly estimate the size of a network with probability one. If *t*_max_ could be exactly determined for the network, we would be absolutely sure that each residue message has reached every node and hence, each node is aware of the size of the network. This would be in direct violation of the aforementioned result. However, depending on the prior knowledge about the network, various estimates of *t*_max_ can be made as follows. In Sec. Theorems and Proofs (Corollary 1) of the S1 File, we show that the maximum time required for all nodes to reach the final state, i.e., the inactive state, has the above boundary of *t*_*r*_ = 3*N* + 2. It is also trivial that the number of time steps required to broadcast a message across a network of size *N* is, in the worst-case, *t*_*b*_ = *N* − 1. Therefore, *t*_max_ is bounded above by *t*_*r*_ + *t*_*b*_ = 4*N* + 1. Hence, if an overestimate *N*_max_ of the network size is known apriori, we can set *t*_max_ = 4*N*_max_ + 1 to know the exact size of the network in finite time.

### Remarks on the AnB algorithm

As shown in Fig 1, a node spends exactly one iterative step as a leaf, and at most one iterative step as a residue node. Therefore, a typical node spends most of its iterative steps in either active or inactive states.

We can now elaborate on the similarities and differences between the proposed AnB algorithm and the standard node-counting method on a tree network which were indicated earlier. On comparion, we note the following points of interest.

1. Nodes in a tree network can also be classified into four categories analogous to those in the AnB algorithm: (a) the root (similar to *s*_*i*_ = *R*), (b) leaves (similar to *s*_*i*_ = *L*), (c) pruned leaves (similar to *s*_*i*_ = *I*) and (d) other nodes still in the network (similar to *s*_*i*_ = *A*).
2. In a tree network, leaves are easily identified as nodes with degree one. Since this is not true for a general network, we use the condition in Line 1 to identify, at each iteration step, the nodes which are to be labeled as leaves.
3. After a node has been identified as a leaf in a tree network, it passes on its local count to its parent and gets transformed to a pruned leaf. In a tree network, the parent of each node is unique. However, in a general network, a leaf node may have more than one parent. Therefore, in the AnB algorithm, the local count of each leaf is divided equally among all parents to avoid over-counting number of nodes.
4. Once the counts have been passed on, the leaf node becomes an inactive node, similar to the pruned leaves in a tree network. If there are no active neighbors (‘parents’) to which a node can pass on its local count, it becomes a residue node, which is similar to the root of the tree. While the structure of the tree implies that there can be only one root of a tree, there is no such restriction for a general network. Hence, the count of the size of a general network gets concentrated into the residue nodes which is then broadcast and recombined in the final stages of the AnB algorithm.

It is to be noted that each node checks for the reception of a message of type *h* = broadcast at each iteration. This is necessary because messages of type *h* = broadcast carry the node count of a part of the network as counted by a residue node. Therefore, all nodes which receive such a message should add it to their final count and send it further. This is in contrast with the other types of messages which are intended only for nodes in active or (as in case of *h* = leaf) leaf states.

## Analysis of the algorithm

In this section, we demonstrate the correctness of the AnB algorithm and analyze the algorithm performance in terms of time, communication, and memory costs against the known node-counting algorithms. We do not compare AnB with stochastic algorithms which only compute an estimate of the network size that increases over time, but we limit our comparison against algorithms that return the exact node count in a finite time: the All-2-All algorithm and the Single Tree (ST) algorithm [18].

The All-2-All algorithm is, to the best of our knowledge, the only known deterministic algorithm for node counting which can work on any type of connected network regardless of its topology. In the All-2-All algorithm, each node broadcasts its id, and all received ids, to all its neighbors and every node counts the number of received unique ids.

The ST algorithm, instead, is the most efficient of the three algorithms proposed by Bawa et al. in [18]. Despite being stochastic, the ST algorithm is proved to return the exact network size in a finite time. The ST algorithm, similarly to AnB, relies on the construction of a tree-like hierarchy. However, in its original form, the ST algorithm allows only a single node to compute the network size. In order to allow all the nodes of the network to know the network size, the ST algorithm can be extended in the following two ways: (a) one randomly selected node executes the ST algorithm and then broadcasts the computed size to all other nodes; or (b) all the nodes of the network simultaneously execute the ST algorithm and compute the network size independently. Employing alternative (a) requires the nodes to be able to select in a decentralized way which node will execute the ST algorithm. Decentralized node-selection adds a new problem which may require further assumptions on the network topology or on the initial knowledge of the nodes [31]. Therefore, in our comparison against the ST algorithm, we employ alternative (b) by which every node makes an independent count of the network size.

We provide a comparison both as worst-case algorithm complexity and with generic analytical equations for each type of cost. When such analytical solutions are not possible, we provide the results of numerical simulations for specific graph topologies. In fact, the AnB algorithm is proved to work on any connected graph regardless on the graph topology. Through our analysis, we highlight the differences in performance for each topology.

### Correctness of the AnB algorithm

In Sec. Theorems and Proofs of the S1 File, a detailed proof of correctness of the algorithm is provided. A brief sketch of the proof is as follows. We begin by identifying a sequence of time steps of the algorithm when the variables *e*_*i*_ and $E_{i}$ correctly give correct information about the neighborhood of the node *i* (see Theorem 1). We say that, at these time steps, the network is in the *resting state*. We then show that, as the network progresses from one resting state to another, the number of active states decreases. During this process, the information about their local node counts *c*_*i*_ gets concentrated into the nodes which pass through the residue state (see Theorem 2). Therefore, when no active nodes are present in the the network, the information about the size of the network is concentrated in the nodes which passed through the residue state. This information is then broadcast throughout the network and is accumulated by each node (see Theorem 3).

### Comparison with other algorithms in terms of complexity

We compare the efficiency of the AnB algorithm against the All-2-All and the Single Tree (ST, [18]) algorithms in terms of three aspects: (a) the time required to compute the network size by every node, (b) the number of messages sent by all nodes (i.e. the communication cost), and (c) the minimum amount of memory required by each node to execute the algorithm (i.e. the memory cost).

Note that, it is difficult to compare the efficiency of AnB against most other stochastic algorithms because their efficiency depends on the desired accuracy of the results. The more accurate we want the results to be, the longer the stochastic algorithms should run, at the cost of increased time and/or communication costs. On the other hand, deterministic algorithms like ours give accurate results in a finite time and make possible asymptotic performance analysis.

**Fig 3:**
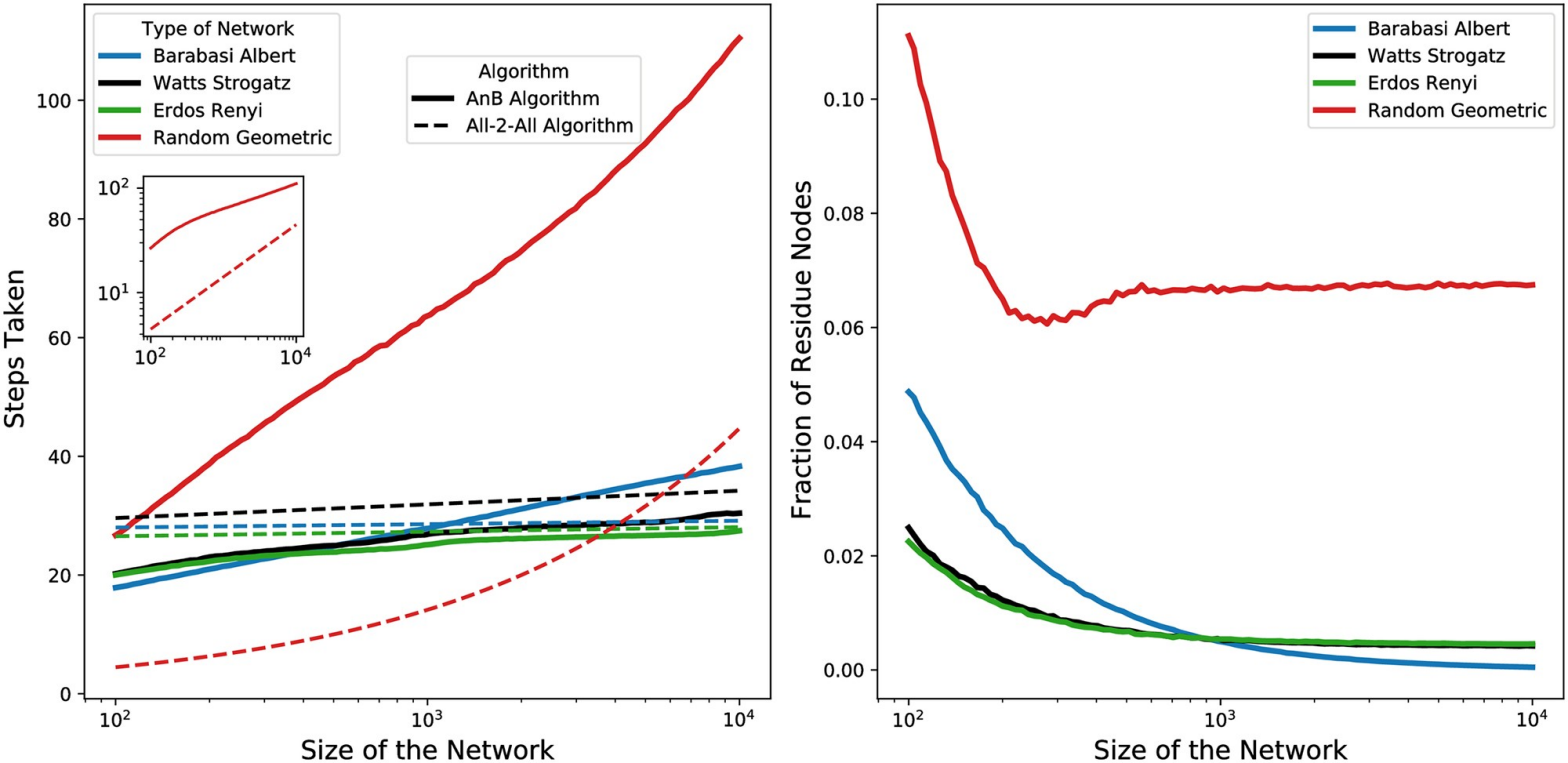
Numerically estimated time costs of the AnB algorithm. The left panel shows, on a log-linear scale, the total number of iterative steps taken by the AnB algorithm for different random networks (solid lines). The dashed lines show the scaling of the time for the All-2-All method, which corresponds to the network diameter *D* from Table 3. The diameter is known up to a scaling factor, here we report curves scaled to values comparable to AnB’s execution time to ease the comparison. In fact, the intersection of same-colour curves indicates that for large networks, the AnB algorithm is asymptotically slower than the All-2-All method. This is the case for all the analyzed network topologies but the Random Geometric networks. In RG networks, All-2-All shows a steeper curve that would slow down the process for very large networks (see inset on a log-log scale). The right panel shows the fraction of residue nodes $x=\frac{r}{N}$ in the network. Low *x* implies low *r* and hence better performance of AnB algorithm in terms of memory and communications cost (see Table 2). For each network size, we report the average results for the simulation of 1,000 independent random networks. (95% confidence intervals are reported in the left panel as shades but often are smaller than the line width).

The efficiency results for the AnB algorithm are derived in Sec. Complexity Analysis of the S1 File and reported in Table 2. We derive exact results for the communication and memory costs. Instead, computing a precise equation of the time cost is difficult, as it depends strongly on the topology of the network which evolves at every time step (see discussion in Sec. Time Cost). Through Theorem 3 in Sec. Theorems and Proofs, we computed the upper bound of the time complexity of AnB. To analyse the exact performance in terms of time, instead, we computed a set of numerical simulations on various graph topologies whose results are shown in Fig 3. In particular, we implemented and tested the AnB algorithm on four different types of random networks as listed in Table 3. The results of our analysis show a qualitative difference in algorithm performance as a function of the network topology. We employed these numerical simulations to compare the temporal performance of AnB with the All-2-All algorithm and to make general considerations on the execution time of the AnB algorithm (see also Sec. Time Cost).

**Table 2:** Exact costs for the two algorithms for a general network with diameter *D*, average degree *d*, and *r* residue nodes. For memory cost, we indicate the individual degree *d*_*i*_ for the generic node *i*. The AnB algorithm is more efficient than the All-2-All and the ST methods in terms of memory and communication. Analytical solution for time is out of reach and we provide numerical results in Fig 3.

Algorithm	Time	Communication	Memory
AnB	numerically in Fig 3	*N*(4 + *r* + *d*) − *r*	(2*d*_*i*_ + *r* + 5)log(*N*)
All-2-All	*D*	*N* ^2^	*N* log(*N*)
ST	2*D*	2*N*^2^	2*N* log(*N*) + *d*_*i*_ *N*

**Table 3:** The analyzed networks.

Type of network	Constructing algorithm	Parameters	Diameter *D*
Scale-free	Barabasí Albert [32]	*m* = 10	$\frac{\text{log}\;N}{\text{log}\;\text{log}\;N}$ , [33]
Random	Erdös Renyí [34]	$p_{e}=\frac{20}{N}$	$\frac{\text{log}\;N}{\text{log}\;(p_{e}N)}$ , [35]
Small-world	Watts Strogatz [36]	*k* = 20, *p*_*r*_ = 0.5	log *N*, [33]
Random Geometric	Penrose [37]	$r=\sqrt{\frac{10}{N}}$	$\frac{\sqrt{2}}{r}$ , [38]

Description of the internal parameters: *m*: Number of edges with which a new node attaches to existing nodes; *p*_*e*_: Probability of forming an edge; *k*: Number of nearest neighbors to which the node initially connects; *p*_*r*_: Rewiring probability; *r*: Threshold distance unto which two nodes are connected.

The time, communication, and memory costs for All-2-All algorithm are relatively easy to compute. In terms of time, the algorithm ends when the messages created by every node (containing its id) reach every other node. Therefore, the time steps required for this to happen is equal to the diameter *D* of the network. In terms of communication, since each node broadcasts the id of every node to its neighborhood, the number of messages sent by each node is *N* and hence the total number of messages sent in the whole network is *N*^2^. Finally, in terms of memory, each node needs to store the id of every node in the network. Therefore, the minimum memory required by each node is *N* log(*N*), by assuming that each id needs at least log(*N*) bits.

The time and communication efficiency of the ST algorithm has been outlined by Bawa et al. in [18]. We updated their efficiency measures in order to include the changes required to allow all nodes to compute the network size. Additionally, we derived the memory cost which was not originally indicated in [18]. The details of the complexity analysis are reported in Sec. Complexity Analysis of the S1 File; the results are reported in Table 2.

**Fig 4:**
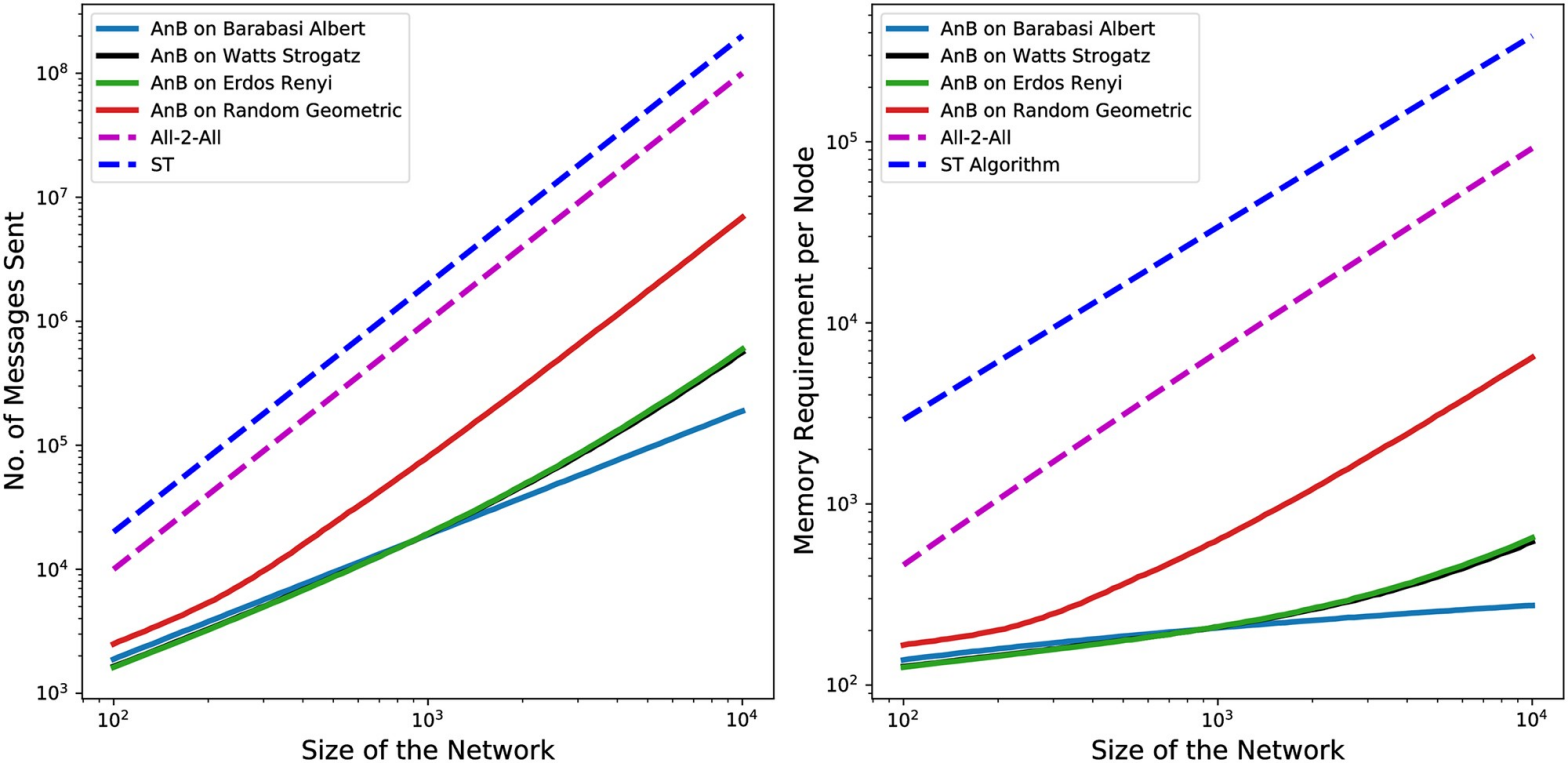
The AnB is the most efficient algorithm in terms of communication and memory costs, compared with the All-2-All and ST algorithms. The left panel shows the total number of messages sent by the nodes. The right panel shows the corresponding memory requirements per node with average connectivity degree *d*. In both panels, the dashed lines show the scaling for the All-2-All and ST algorithms, whereas the solid lines of various colors show the scaling for the AnB algorithm. Note that the number of messages sent and the memory requirements depends only on the network size for All-2All and ST algorithms and hence, are independent of the network topology. However, the number of messages sent and the memory requirements for AnB algorithm depends on the number of residue nodes which in turn depends on the topology of the network. Therefore, their dependence on the network topology is also explicitly shown.

The results in Table 2 show that the AnB algorithm has the lowest costs in terms of memory and computation compared with the All-2-All and ST algorithms (see also Fig 4). The efficiency of the AnB algoritms is higher for networks which have the number of ‘residue’ nodes *r* much smaller than *N*. This is the case for most random networks as shown in Fig 3 (right panel). Our analysis also shows that the largest share of communication messages are typically sent by the residue nodes and the largest memory is typically required to store the ids of the residue nodes. Since the fraction of residue nodes is low for all the analyzed network classes, with the AnB algorithm the nodes send comparatively fewer messages and have lower memory requirements than with the All-2-All and ST algorithms. The only cases where the All-2-All and ST algorithms might perform better than AnB in terms of memory and communication are completely connected networks, almost completely connected networks, and networks with specific topologies (such as ring networks; see detailed discussion in Sec. Performance on Ring and Complete Networks of the S1 File). In terms of time, Fig 3 (left panel) shows that the All-2-All method scales as the network diameter *D* and the AnB algorithm has comparable, or slightly worse, time performance. Finally, in terms of all three complexity aspects (time, communication, and memory), in the worst case (i.e., when *d*_*i*_ = *N* − 1 and *r* = *N*), the AnB algorithm has an asymptotically complexity equal to the other algorithms (see Table 4 in the S1 File). Therefore, we conclude that the AnB algorithm is advantageous for applications with constrained or high-cost communication and memory, as confirmed by the results reported in Table 2 and Fig 4.

## Conclusion

In this paper, we propose the AnB algorithm, a deterministic algorithm by which all nodes of a network can become aware of its size. The AnB algorithm assumes no inherent hierarchy among the nodes and no prior knowledge of the network topology. Instead, it depends on (a) the nodes having unique ids and (b) the nodes being able to communicate with its immediate neighbors. We also analyze the efficiency of the AnB algorithm and compare it against the known algorithms. We conclude that the AnB algorithm is significantly better than the known deterministic algorithms on average in terms of memory and communication costs. This has potential benefits in engineering where decentralized systems composed of a large number of units that operate without a central controller are spreading in various application domains, since they can offer scalable, cost-effective, robust solutions. Three examples of such domains are swarm robotics [39], internet of things [40], and wireless sensor networks [41].

In this concluding section, we outline some of the salient features of the AnB algorithm and the ways in which it can be extended and applied to various physical systems.

1. **Quorum sensing:** It is notable that the local node counter *c*_*i*_ and the final count variable *n*_*i*_ are monotonic functions of time. Since both variables are aggregates of the size of the network, max(*c*_*i*_, *n*_*i*_) gives a lower bound of the network size at any point in time. This can be useful in systems which are trying to determine if a quorum is present on not [42]. Since in these cases the system is trying to determine if the network size is above a certain threshold or not, a node *i* can enter the broadcast phase as soon as *c*_*i*_ is greater than the threshold and inform the other nodes of the quorum being reached.
2. **Spontaneous hierarchy creation:** While the AnB algorithm assumes no hierarchy among the nodes, the progression of the algorithm can be used to create it depending on the time when a node enters the broadcast phase. If a node enters the broadcasting phase late, it is more likely to be connected to nodes with high degrees, and hence be more ‘central’. Conversely, if a node enters the broadcasting phase earlier, it is more likely to be ‘peripheral’. While various other centrality measures exist for such classification of nodes in a network (for instance, closeness centrality [43] and betweenness centrality [44]), they generally require the computation and ordering of a measure by a centralized agency. In the proposed AnB algorithm, the nodes can spontaneously organize themselves into a hierarchy.
3. **Computation of other aggregate quantities:** Similar to other previously known algorithms of network size estimation [5, 18], the AnB algorithm can also be used to compute other global properties across networks. For example, if each node *i* is associated with a property *s*_*i*_, they can compute the sum ∑*s*_*i*_ by simply setting *c*_*i*_ = *s*_*i*_ and executing the AnB algorithm. Similarly, other aggregate quantities such as averages and maximums/minimums can also be computed by suitably adopting the AnB algorithm.

## Supporting information

S1 File (PDF)
