# Supplementary material for: Memory and communication efficient algorithm for decentralized counting of nodes in networks
Source: PLoS One. 2021 Nov 22;16(11):e0259736. doi: 10.1371/journal.pone.0259736 (PMC8608303; doi:10.1371/journal.pone.0259736)
Supplement: S1 File — (PDF) [file pone.0259736.s001.pdf]

Supporting Information
for
Memory and communication efficient algorithm for decentralized counting of nodes in networks

Arindam Saha[1], James A. R. Marshall[1], Andreagiovanni Reina[1,2]
1 Department of Computer Science, University of Sheffield, S1 4DP, UK

2 IRIDIA, Université Libre de Bruxelles, Belgium

Theorems and Proofs

Let $\mathcal{G}$ be a connected, undirected and unweighted network whose size is to be determined by its nodes i using the AnB algorithm. Let $N > 0$ be the size of $\mathcal{G}$. Also, let $\mathcal{A}(t)$, $\mathcal{B}(t)$ and $\mathcal{C}(t)$ be the sets of nodes in active, leaf and residue states respectively at iteration time step t. Let $N_A(t)$, $N_B(t)$ and $N_C(t)$ be the number of elements of $\mathcal{A}(t)$, $\mathcal{B}(t)$ and $\mathcal{C}(t)$, respectively. Let $m_i(t)$ be the number of messages of type $h = 4$ received by node $i \in \mathcal{G}$ at iteration time step t. Finally, let Γ_i be the total number of neighbors of node i and and $\tilde{\Gamma}_i(t)$ be the number of neighbors of the node i which are also in $\mathcal{A}(t)$.

Let us first present the following trivial properties of these sets.

Result 1. *The following statements are true for any network $\mathcal{G}$ evolving under Algorithm 1:*

(a) $\mathcal{A}(t)$ can be partitioned as

$$\mathcal{A}(t) = \mathcal{A}(t+1) \cup \mathcal{B}(t+1). \tag{1}$$

(b) $\mathcal{B}(t)$ and $\mathcal{C}(t)$ are related as follows,

$$\mathcal{C}(t+1) \subseteq \mathcal{B}(t). \tag{2}$$

(c) From Eqs. 1 and 2 we have,

$$\mathcal{C}(t+2) \subseteq \mathcal{B}(t+1) \subseteq \mathcal{A}(t). \tag{3}$$

Definition 1. *Let the network $\mathcal{G}$ said to be in resting state at iteration time step $t \geq 0$ if the following are true:*

$$m_i(t) = 0 \quad \forall i \in \mathcal{G}, \tag{4}$$

$$N_B(t) = 0. \tag{5}$$

Let the ordered sequence of all iteration time steps $t \geq 0$ when the network $\mathcal{G}$ is in resting state be denoted by (T_n) where $n = \{0, 1, 2, \ldots\}$.

Using this definition, we state and prove the following results.

Lemma 1. *The network $\mathcal{G}$ is in resting state at iteration time step $t = 0$. In other words $T_0 = 0$.*

Proof. Prior to setting $t = 0$ in Line 9, all nodes are set to be in active state (Line 1) and they send no messages of type $h = 4$. Therefore no nodes can receive any such message at $t = 0$. Hence, Eqs. 4 and 5 are satisfied at $t = 0$. □

Lemma 2. *If $\mathcal{G}$ is in resting state at $t = T_n$ and $\mathcal{A}(T_n) = \emptyset$, then $T_{n+1} = T_n + 1$.*

Proof. The network $\mathcal{G}$ being in resting state at $t = T_n$ implies $N_B(T_n) = 0$. Hence, no message of type $h =$ count is sent at $t = T_n$. Therefore, $m_i(T_n + 1) = 0 \ \forall \ i \in \mathcal{G}$. Also, since $\mathcal{A}(T_n) = \emptyset$ therefore, due to Eq. 3, $\mathcal{B}(T_n + 1) \subseteq \mathcal{A}(T_n) = \emptyset$. Hence, the network $\mathcal{G}$ is in the resting state at $T_{n+1} = T_n + 1$. □

Theorem 1. *For all iteration time steps $t = T_n$ when $\mathcal{G}$ is in resting state and $N_A(T_n) > 0$, the following statements are true for any node $i \in \mathcal{A}(T_n)$:*

(a) The variable $e_i(T_n) = \tilde{\Gamma}_i(T_n)$.

(b) *For each active node j in the neighborhood of i, there is a corresponding element $(j, e_j) \in \mathcal{E}_i(T_n)$ such that $e_j = \tilde{\Gamma}_j(T_n)$.*

Proof. We will prove this theorem by induction.

- **Base case:** From Lemma 1 we know that $\mathcal{G}$ is in resting state at $t = 0$. Prior to setting $t = 0$, the variable $e_i(0)$ is initialized by counting the number of messages of type $h =$ echo received. Since this message is sent by all nodes of the network and the message travels to all nodes in the neighborhood therefore, $e_i(0) = \Gamma_i = \tilde{\Gamma}_i(0)$, since all nodes are in active state at $t = 0$. Thereafter, these counts are sent to the entire neighborhood as messages of type $h =$ degree. The set $\mathcal{E}_i(0)$ is constructed by the accumulating all such messages received. Therefore, for each $(j, e_j) \in \mathcal{E}_i(0)$, we have $e_j = \Gamma_j = \tilde{\Gamma}_j(0)$.

- **Inductive step:** Let us assume that the theorem is true for some iteration time step $t = T_k$ when $\mathcal{G}$ is in resting state. Let $T_{k+1} > T_k$ be the next iteration time steps when $\mathcal{G}$ is in resting state. In order to prove that the theorem is true for $t = T_{k+1}$, we show the following in sequence:

  (A) $T_{k+1} > T_k + 1$ and both parts (a) and (b) of the theorem are false at $t = T_k + 1$.

  (B) If $\mathcal{A}(T_k + 1) = \emptyset$ then $T_{k+1} = T_k + 2$ and the theorem is vacuously true, otherwise, if $\mathcal{A}(T_k + 1) \neq \emptyset$:

    (i) $T_{k+1} > T_k + 2$ and part (a) of the theorem is true and part (b) is false at $t = T_k + 2$.

    (ii) $T_{k+1} = T_k + 3$ and both parts (a) and (b) of the theorem are true at $t = T_k + 3$.

  Thereby completing the inductive step.

  Let us consider a node $i \in \mathcal{A}(T_k)$ such that $e_i = \min\{e_j(T_k) : j \in \mathcal{A}(T_k)\}$. All such nodes trivially satisfy the inequality in Line 19. Also, since $\mathcal{G}$ is in resting state, then $m_i(T_k) = 0$. Therefore, the condition stated in Line 19 is true for i, which in turn implies that i sends a message of the form $m_{i,\text{leaf}}$ at iteration time step T_k and would enter the leaf state in the next iteration time step. Therefore,

$$N_B\left(T_k + 1\right) > 0. \tag{6}$$

(A) **Since** $N_B(T_k + 1) \neq 0$**, we can say that** $T_{k+1} > T_k + 1$**.** At iteration time step $T_k + 1$, the set $\mathcal{A}(T_k + 1)$ can be partitioned into three subsets:

$$\mathcal{A}(T_k + 1) = \mathcal{A}_0(T_k + 1) \cup \mathcal{A}'(T_k + 1) \cup \mathcal{A}''(T_k + 1) \tag{7}$$

where $\mathcal{A}'(T_k+1)$ is the set of active neighbors of nodes in $\mathcal{B}(T_k+1)$, $\mathcal{A}''(T_k + 1)$ is the set of active neighbors of nodes in $\mathcal{A}'(T_k + 1)$ which are not already in $\mathcal{A}'(T_k + 1)$, and $\mathcal{A}_0(T_k + 1)$ is the set of all other active nodes in $\mathcal{A}(T_k + 1)$.

Consider the value of $e_l(T_k + 1)\ \forall\ l \in \mathcal{A}'(T_k + 1)$. It has not yet changed since $t = T_k$. However, the actual value of $\tilde{\Gamma}_l(T_k + 1)$ has changed. Therefore, **part (a) of the lemma is false at** $t = T_k + 1$**.**

Similarly, consider the elements of $\mathcal{E}_m(T_k + 1)\ \forall\ m \in \mathcal{A}''(T_k + 1)$. Since each element in $\mathcal{A}''$ has an active neighbor in $\mathcal{A}'$, there exists at least one $(l, e_l) \in \mathcal{E}_m(T_k + 1)$ such that $l \in \mathcal{A}'$. Since this e_l does not represent the current value of $\tilde{\Gamma}_l$ **therefore, part (b) of the lemma is also false at** $t = T_k + 1$**.**

Note that, the active neighbors of all nodes $p \in \mathcal{A}_0(T_k + 1)$ are in the set $\mathcal{A}''(T_k + 1)$. Since, nodes in $\mathcal{A}''(T_k + 1)$ are still in the active state, therefore, $e_p = \tilde{\Gamma}_p(T_k + 1)$ for all $p \in \mathcal{A}_0(T_k + 1)$. Furthermore, the neighbors of nodes in $\mathcal{A}''(T_k+1)$ are in $\mathcal{A}'(T_k+1)$. Since all nodes in $\mathcal{A}'(T_k + 1)$ are in active state, the number of active neighbors of nodes in $\mathcal{A}''(T_k+1)$ has not changed. Therefore each element $(m, e_m) \in \mathcal{E}_p(T_k + 1)$ still accurately represents the active neighbors m of p and the number of active neighbors e_m of m.

The number of messages of type h = leaf received by a node $b \in \mathcal{B}(T_k + 1)$ gives the number of its neighbors which were active at $t = T_k$ but have transitioned to the leaf state at $t = T_k + 1$. Note that $e_b(T_k) = \Gamma_b(T_k)$. Since $e_b(T_k + 1)$ is obtained by taking the difference between $e_b(T_k)$ and the aforementioned number of messages therefore, it gives the number of neighbors of b which are still in the active state at $t = T_k + 1$. In other words,

$$e_b(T_k + 1) = \tilde{\Gamma}_b(T_k + 1) \tag{8}$$

If $e_b(T_k+1) = 0$, b enters the residue state at $t = T_k+1$; otherwise, it sends a message $m_{b,\text{count}}$ and enters the inactive state.

Finally, since no message of type h = count or h = reduce are received by any node, we can say that $e_i(T_k + 1) = e_i(T_k)$ and $\mathcal{E}_i(T_k+1) = \mathcal{E}_i(T_k)$. Therefore, no active nodes become leaf nodes. Hence,

$$\mathcal{A}(T_k + 2) = \mathcal{A}(T_k + 1) \tag{9}$$

and

$$\mathcal{B}(T_k + 2) = \emptyset. \tag{10}$$

(B) We can assume two mutually exclusive cases: $\mathcal{A}(T_k + 1) = \emptyset$ or $\mathcal{A}(T_k + 1) \neq \emptyset$.

If we assume $\mathcal{A}(T_k + 1) = \emptyset$, from Eq. 1, $\mathcal{A}(T_k + 1) = \emptyset$ implies $\mathcal{B}(T_k+2) = \emptyset$. Also from Eq. 7, $\mathcal{A}(T_k+1) = \emptyset$ implies $\mathcal{A}'(T_k+1) = \emptyset$. Therefore, from Eq. 8, we have $e_b(T_k + 1) = 0 \; \forall \; b \in \mathcal{B}(T_k + 1)$ which in turn implies $m_i(T_k+2) = 0$. Hence, $\mathcal{A}(T_k+1) = \emptyset$ implies that the network is in resting state at $T_{k+1} = T_k + 2$. From Eq. 9 we have, $\mathcal{A}(T_{k+1}) = \mathcal{A}(T_k + 2) = \mathcal{A}(T_k + 1) = \emptyset$ also implies that the hypothesis of the theorem is false at iteration time step $t = T_{k+1}$. Therefore, the statement of the theorem is vacuously true.

For the remaining part of the proof, we assume that,

$$\mathcal{A}(T_k + 1) \neq \emptyset. \tag{11}$$

(i) The messages $m_{b,\text{count}}$ are received by all neighbors of $b \in \mathcal{B}(T_k + 1)$ at $t = T_k + 2$. **This proves that the network is not in the resting state at $t = T_k + 2$.** On receiving each such message, the active nodes $l \in \mathcal{A}'(T_k + 1)$ remove the element (b, e_b) from $\mathcal{E}_l(T_k + 2)$ and decrease the value of $e_l(T_k+2)$ by one. The total number of such messages received by l at $t = T_k + 2$ is equal to reduction in $\tilde{\Gamma}_l$ from $t = T_k$ to $t = T_k + 1$. Furthermore, since $\mathcal{A}(T_k + 2) = \mathcal{A}(T_k + 1)$, therefore, $\tilde{\Gamma}_l$ has not changed from $t = T_k + 1$ to $t = T_k + 2$. Therefore, $e_l(T_k + 2) = \tilde{\Gamma}_l(T_k + 2) \; \forall l \in A'(T_k + 1)$. The $e_{l'}(T_k+2)$ of all other nodes $l' \in \mathcal{A}(T_k+3) \setminus A'(T_k+1)$ gives the actual number of their active neighbors anyways. **Therefore, part (a) of the theorem is true at $t = T_k + 2$.**

However, the nodes $m \in \mathcal{A}''(T_k + 1)$ remain unaware of the changes in number of active neighbors of the nodes in $\mathcal{A}'(T_k+$

1). **Hence, part (b) of the theorem is still false at** $t = T_k + 2$ because the value of e_l stored in $(l, e_l) \in \mathcal{E}_m(T_k + 2)$ is still equal to $e_l(T_k)$ and not to $e_l(T_k + 2)$. In order to notify the nodes $m \in \mathcal{A}''(T_k + 1)$ of the changes of $e_l(T_k)$, nodes $l \in \mathcal{A}'(T_k + 2)$ send a message $m_{l,\text{reduce}}$ for each $m_{b,\text{count}}$ received.

Finally, since any node $i \in \mathcal{A}(T_k + 2)$ which has changed $e_i(T_k + 1)$ has also received a message of type $h = \text{count}$, it does not change its state. Any other node in $\mathcal{A}(T_k + 2)$ can also not change its state due to arguments similar to the ones presented in (A). Therefore,

$$\mathcal{A}(T_k + 3) = \mathcal{A}(T_k + 2) \tag{12}$$

and

$$\mathcal{B}(T_k + 3) = \emptyset. \tag{13}$$

(ii) Eq. 10 implies that there were no messages $m_{b,\text{count}}$ sent at iteration time step $T_k + 2$. Therefore,

$$m_i(T_k + 3) = 0 \;\forall\; i \in \mathcal{G}. \tag{14}$$

This, when combined with Eq. 13 implies $\mathcal{G}$ is in the resting state at $T_k + 3$.

Eq. 14 also implies that $e_i(T_k + 3) = e_i(T_k + 2) \;\forall\; i \in \mathcal{G}$. **Therefore, part (a) of the theorem still holds at $t = T_k + 3$.**

Now, let us consider the messages $m_{l,\text{reduce}}$ sent by all nodes $l \in \mathcal{A}'(T_k + 1)$. On receiving each such message, the nodes $m \in \mathcal{A}''(T_k + 1)$ update $(l, e_l) \in \mathcal{E}_m(T_k + 2)$ to $(l, e_l - 1)$. Since the number of messages received equals the reduction in the degree of node l, after receiving all the messages $m_{l,\text{reduce}}$, the tuple (l, e_l) gives the correct number e_l of active neighbors of l. **Therefore, part (b) of the theorem is satisfied.**

Hence, if we assume the Theorem 1 to be true at some $t = T_k$, it is also true for another $T_{k+1} > T_k$. We have $T_{k+1} = T_k + 2$ if there are no active nodes left at $T_k + 1$ (i.e. $\mathcal{A}(T_k + 1) = \emptyset$); otherwise, if $\mathcal{A}(T_k + 1) = \emptyset$, we have $T_{k+1} = T_k + 3$. Since we already know that the theorem is true for $t = T_0 = 0$, it is true for all T_n by induction. □

From Lemma 2 and Theorem 1, we deduce the following results for resting iteration time steps T_n.

Result 2. *For any T_n,*

$$T_{n+1} \leq T_n + 3. \tag{15}$$

Result 3. *If $N_A(T_n) = 0$, then*

$$N_A(T_{n+1}) = N_A(T_n) = 0. \tag{16}$$

Instead, if $N_A(T_n) > 0$, then

$$N_A(T_{n+1}) < N_A(T_n). \tag{17}$$

Result 4. *If $\mathcal{A}(T_k) \neq \emptyset$, then*

$$\mathcal{A}(T_{k+1}) = \mathcal{A}(T_k + 1) \subset \mathcal{A}(T_k). \tag{18}$$

It directly follows from Lemma 1 and Result 2 that,

$$T_n \leq 3n\,. \tag{19}$$

Additionally, the following corollary follows directly from Result 2 and Eq. 19.

Corollary 1. *For a network $\mathcal{G}$ of size $N > 0$ evolving under Algorithm 1, there exists a iteration time step $t_R \leq 3N$ such that $N_A(t_R) = 0$.*

Proof. Let us assume that $N_A(t) > 0$ for all $t \leq 3N$. This implies,

$$N_A(3N) > 0 \tag{20}$$

Since $N_A(t)$ is a monotonically decreasing function of iteration time step and $T_N \leq 3N$ (due to Eq. 19), we can say that,

$$N_A(T_N) > 0. \tag{21}$$

Note that $N_A(t)$ can change only is steps of one since it is a non-negative integer function. Therefore, using Eq. 17,

$$N_A(T_N) \leq N_A(T_{N-i}) - i \tag{22}$$

for any positive integer $i \leq N$. Setting $i = N$, we get,

$$N_A(T_N) \leq 0 \tag{23}$$

since $T_0 = 0$ and $N_A(0) = N$ due to all nodes of the network being active at $t = 0$. This is a direct contradiction to Eq. 21. Therefore, our assumption was wrong, hence proving the corollary. □

Corollary 2. *The number of residue nodes* $N_C(t) \geq 0$ *only if* $t = T_n + 2$ *for some* n *and* $N_A(T_n) > 0$*. Otherwise,* $N_C(t) = 0$*.*

Proof. It directly follows from Eqs. 6, 10 and 13 that if $N_A(T_n) > 0$, nodes in leaf state are present only at $t = T_n+1$. This, in turn, implies $N_C(t) \geq 0$ only if $t = T_n + 2$ due to Eq. 3. If there are no active nodes present at T_n, then it is obvious that there would be no leaf or residue nodes in the future. □

Definition 2. *Let the cumulative residue set of a network* $\mathcal{G}$ *at iteration time step* t *be defined as,*

$$\mathcal{P}(t) = \bigcup_{i=0}^{t} \mathcal{C}(i). \tag{24}$$

Definition 3. *Let the cumulative residue index of a network* $\mathcal{G}$ *at iteration time step* t *be defined as the number of elements in* $\mathcal{P}(t)$*,*

$$r(t) = |\mathcal{P}(t)|\,. \tag{25}$$

Clearly, $r(t)$ is a non-negative, non-decreasing integer function of t. We now prove the following corollary regarding $r(t)$.

Corollary 3. *If* T_R *is the iteration time step when the network* $\mathcal{G}$ *is the resting state,* $N_A(T_R) = 0$*, and* $N_A(T_{R-1}) > 0$*, then* $r(T_R) > 0$*.*

Proof. If $r(T_{R-1}) > 0$, then the corollary is trivially proved. Therefore, let us consider the case when $r(T_{R-1}) = 0$. Since $N_A(T_{R-1}) > 0$, we can use Result 4 to get $\mathcal{A}(T_{R-1}{+}1) = \mathcal{A}(T_R) = \emptyset$. This in turn implies $\mathcal{B}(T_{R-1}{+}1) = \mathcal{A}(T_{R-1})$ due to Eq. 1. This leads to the situation where, at $t = T_{R-1} + 1$ none of the leaf nodes have any active neighbors. Therefore, due to Eq. 8,

$$e_b(T_{R-1} + 1) = 0 \quad \forall b \in \mathcal{B}(T_{R-1} + 1). \tag{26}$$

Hence, the condition in Line 26 of the Algorithm 1 is satisfied for all nodes $b \in \mathcal{B}(T_{R-1} + 1)$, which become residue nodes. Thus, from Theorem 1, because $N_A(T_R) = 0$, we have

$$\mathcal{C}(T_{R-1} + 2) = \mathcal{C}(T_R) = \mathcal{B}(T_{R-1} + 1) = \mathcal{A}(T_{R-1}) \tag{27}$$

Hence, $\mathcal{C}(T_R) \neq \emptyset$ and therefore $r(T_R) > 0$. □

Definition 4. *Let the overall count $I(t)$ be defined as,*

$$I(t) = \sum_{i \in \mathcal{Q}(t)} c_i(t) \tag{28}$$

where $\mathcal{Q}(t) = \mathcal{A}(t) \cup \mathcal{P}(t)$.

Theorem 2. *For any iteration time step T_n when $\mathcal{G}$ is in resting state,*

$$I(T_n) = N \tag{29}$$

when N is the size of $\mathcal{G}$.

Proof. We prove this by induction. At $t = T_0 = 0$, the set $\mathcal{A}(t)$ contains all nodes of the network. Since $c_i(t)$ is initialized as $c_i(0) = 1 \; \forall \; i \in \mathcal{G}$, therefore, $\mathcal{Q}(T_0) = \mathcal{Q}(0) = N$.

Now, let us assume that the theorem is true for some iteration time step $t = T_k$ such that $\mathcal{A}(T_k) \neq \emptyset$. Now, by definition,

$$\mathcal{Q}(T_k) = \mathcal{A}(T_k) \cup \mathcal{P}(T_k). \tag{30}$$

Now, since $\mathcal{A}(T_k)$ and $\mathcal{P}(T_k)$ are disjoint sets, we have,

$$I(T_k) = \sum_{a \in \mathcal{A}(T_k)} c_a(T_k) + \sum_{p \in \mathcal{P}(T_k)} c_p(T_k) = N. \tag{31}$$

Let us now consider the network at $t = T_{k+1}$. From Eqs. 1 and 18 we have,

$$\mathcal{A}(T_k) = \mathcal{A}(T_k + 1) = \mathcal{A}(T_k) \setminus \mathcal{B}(T_k + 1). \tag{32}$$

Also due to Corollary 3, we have,

$$\mathcal{P}(T_{k+1}) = \mathcal{P}(T_k) \cup \mathcal{C}(T_k + 2). \tag{33}$$

Therefore,

$$\mathcal{Q}(T_{k+1}) = \mathcal{A}(T_{k+1}) \cup \mathcal{P}(T_{k+1}) = [\mathcal{A}(T_k) \setminus \mathcal{B}(T_k + 1)] \cup [\mathcal{C}(T_k + 2) \cup \mathcal{P}(T_k)] \,. \tag{34}$$

Hence,

$$\mathcal{Q}(T_{k+1}) = [(\mathcal{A}_0(T_k) \cup \mathcal{A}'(T_k + 1)) \setminus (\mathcal{B}'(T_k + 1) \cup \mathcal{C}(T_k + 2))] \cup [\mathcal{C}(T_k + 2) \cup \mathcal{P}(T_k)] \tag{35}$$

where, $\mathcal{A}'(T_k+1)$ is the set of active neighbors at $t = T_k + 1$, $\mathcal{A}_0(T_k)$ is the set of the rest of the nodes in $\mathcal{A}(T_k)$ and $\mathcal{B}'(T_k+1)$ is the set of nodes in $\mathcal{B}(T_k+1)$ which did not become residue nodes.

Now $I(T_{k+1})$ is the sum of $c_i(T_{k+1})$ for all nodes $i \in \mathcal{Q}(T_{k+1})$. Noting that the fourth and the fifth sets in the right hand side of the previous equations are identical, we have,

$$I(T_{k+1}) = \sum_{a_0 \in \mathcal{A}_0(T_k)} c_{a_0}(T_{k+1}) + \sum_{a' \in \mathcal{A}'(T_k+1)} c_{a'}(T_{k+1}) - \sum_{b' \in \mathcal{B}'(T_k+1)} c_{b'}(T_{k+1}) + \sum_{p \in \mathcal{P}(T_k)} c_p(T_{k+1}). \tag{36}$$

Since $c_i(t)$ does not change unless a message of type $h =$ count is received, we have,

$$I(T_{k+1}) = \sum_{a_0 \in \mathcal{A}_0(T_k)} c_{a_0}(T_k) + \sum_{a' \in \mathcal{A}'(T_k+1)} (c_{a'}(T_k) + \delta_{a'}) - \sum_{b' \in \mathcal{B}'(T_k+1)} c_{b'}(T_k) + \sum_{p \in \mathcal{P}(T_k)} c_p(T_k) \tag{37}$$

which can be simplified using Eq. 31 into,

$$I(T_{k+1}) = I(T_k) + \left(\sum_{a' \in \mathcal{A}'(T_k+1)} \delta_{a'} - \sum_{b' \in \mathcal{B}'(T_k+1)} c_{b'}(T_k) \right). \tag{38}$$

Now,

$$\sum_{a' \in \mathcal{A}'(T_k+1)} \delta_{a'} = \sum_{\substack{a' \in \mathcal{A}'(T_k+1) \\ b' \in \mathcal{B}'(T_k+1) \\ a' \text{ is connected to } b'}} \frac{c_{b'}(T_k+1)}{e_{b'}(T_k+1)} \tag{39}$$

$$= \sum_{b' \in \mathcal{B}'(T_k+1)} \tilde{n}_{b'} \left(\frac{c_{b'}(T_k)}{e_{b'}(T_k+1)} \right) \tag{40}$$

$$= \sum_{b' \in \mathcal{B}'(T_k+1)} c_{b'}(T_k) \tag{41}$$

due to Eq. 8. Therefore,

$$I(T_{k+1}) = I(T_k) = N. \tag{42}$$

This proves that the theorem is true for all $T_n \leq T_R$ such that $\mathcal{A}(T_{R-1}) \neq \emptyset$ and $\mathcal{A}(T_R) = \emptyset$. For any $tT_n > T_R$, the overall count $I(T_n)$ still remains invariant, since $\mathcal{A}(T_n)$ continues to be an empty set and hence $\mathcal{P}(T_n)$ does not change further. □

Theorem 3. *There exists some iteration time step $t_{max} \leq 4N + 1$ such that for all $t \geq t_0$,*

$$n_i(t) = N \quad \forall\ i \in \mathcal{G}. \tag{43}$$

Proof. We know from Corollary 1 that for some minimum iteration time step $t_r \leq 3N$, the number of active nodes in the network $\mathcal{G}$ becomes zero. Therefore $\mathcal{A}(t_r) = 0$. Hence, by $t = T_{R+1} = t_r + 2$ all messages of the form $m_{p,\text{broadcast}}$ have been sent by all nodes $p \in \mathcal{P}(T_{R+1})$. The iteration time step required by any such message to reach any other node of the network is $t_b \leq N - 1$. Therefore, by using Theorem 2, we can say that, at iteration time step $t \geq t_r + t_b \leq 4N + 1$, the final count variable for each node $i \in \mathcal{G}$ is,

$$n_i(t) = \sum_{p \in \mathcal{P}(t)} c_p(t) = I(t) = N. \tag{44}$$

□

Complexity Analysis

In this section, we provide the details of the efficiency of the AnB and the ST algorithms with respect to time, communication, and memory costs.

Time Cost

It is difficult to make theoretical estimates about the number of time steps that it takes for the AnB algorithm to work. This is because the number of nodes getting changing states from active to leaf state at each time step depends on the topology of the network. However, the topology of the 'remaining' network evolves as the algorithm progresses as a result of nodes entering into the inactive state. Therefore, while it is possible to estimate the fraction of nodes which get eliminated at the first iteration of the algorithm, estimating the fraction in all subsequent iterations of network reduction is difficult due to difficulties in gauging the changes in the degree distribution and topology of the remaining network. Nevertheless, we can obtain insights

into the time costs of the AnB algorithm by analyzing the results of numerical simulations.

The AnB algorithm can be divided into two distinct phases from the point of view of the network: (a) The network reduction phase: where there are active or leaf nodes still present in the network and the information about the size of the network is being concentrated into a few residue nodes; and (b) The broadcast phase: where no active or leaf nodes are present in the network and the concentrated information is broadcast to all other nodes of the network. Furthermore, since the algorithm successively eliminates nodes with low degrees, the residue nodes which remain after the elimination process are more probable to be nodes with a high degree. In other words, the number of iterations taken in the 'active' and 'inactive' phases depends on the distribution of high/low degree nodes which are determined by the network topology.

Consider a network with a heterogeneous degree distribution such as the Barabási-Albert network, which has few nodes with extremely high degrees and numerous nodes with low degrees. This skewness in degree distribution means that most nodes with low degrees get eliminated without going through the residue state, whereas the few nodes with high degrees become residues. This results in the network having few residue nodes which are well-connected. This makes the broadcast of the counts in residue nodes more efficient. In contrast, consider a network with approximately homogeneous distribution such as the Random Geometric network. Here, since the degrees of all nodes are approximately the same, there is a high probability that there will be a greater number of nodes which go through the residue state.

Having few well connected nodes reduces the number of iterations required in the broadcast phase as the number of residue messages to be sent across the network is low and can be broadcast faster due to high connectivity. That is why networks with more heterogeneous degree distribution, such as the Erdös-Rényi, Barabási-Albert and Watts-Strogatz networks, spend a lower fraction of time in the broadcast phase. In contrast, the Random Geometric network with a more homogeneous degree distribution spends a higher proportion of iterations in the broadcast phase.

Our numerical simulations confirm our insights and show a lower convergence time for heterogeneous networks (see Figure 2 top panel). Our results also show that the time spent in the network reduction phase is also significantly lower in networks with heterogeneous degree distribution. This is

probably due to the fact that an heterogeneous degree distribution allows for a greater number of nodes to be eliminated in one iteration.

Finally, the numerical simulations performed on the different types of random networks (see Figure 2 top panel) reveal that the time taken by the algorithm scales better than $\log N$ for Erdös-Renyí, Barabasí-Albert and Watts-Strogatz networks. This is evident from the sub-linear nature of the plots. On the Random Geometric networks, the algorithm scales worse than $\log N$. Instead, as the broadcast-time required for the network increases relative to the network reduction-time for large networks, the total time required scales as a power of network size N.

Time Cost of the ST Algorithm

The time cost of the ST algorithm when the single node i computes the size of the network depends on the topological location of node i. When all nodes need to compute the ST algorithm the time necessary is exactly $2D$, where D is the network diameter. This is the time necessary to let a message go back and forth throughout the entire network. Therefore the asymptotic worst-case time complexity for the ST algorithm is $\mathcal{O}(D)$.

Communication Cost

To assess the communication cost we compute the expected number of messages to reach convergence, *i.e.* all nodes have the variable n_i equal to the network size. In the proposed algorithm the nodes send various types of messages at various stages of the algorithm. Note that, we assume each message to be 'broadcast' to the neighbors rather than multicast. Therefore, whenever a message is sent from a node to all its neighbors, we count it as a single message. We count the number of messages sent at each stage of the algorithm as follows.

- **Initial count of all neighbors (message type h = echo):** In the initiation phase of the algorithm, each node announces its presence to all its neighbors so that each node becomes aware of its neighborhood. This message is sent once by each node in the network. Therefore, the total number of messages sent in this stage is $M_1 = N$.
- **Broadcasting the initial number of neighbors (message type h = degree):** Each node then broadcasts its number of neighbors.

Since this message is also sent once by each node of the network, the total number of messages sent in this stage is $M_2 = N$.

- **Declaring transition to leaf state (message type h = leaf):** When each node changes its state to $s_i = L$, it broadcasts a message so that any neighboring node in leaf state may update its effective degree. Since the transition from $s_i = A$ to $s_i = L$ is made once by each node, the total number of messages sent in this stage is $M_3 = N$.

- **Declaring transition to inactive state (message type h = count):** Having a network with r residue nodes, the transition from $s_i = L$ to $s_i = I$ is made by $N - r$ nodes. Therefore, the number of messages sent in this stage is $M_4 = N - r$.

- **Updating effective degree (message type h = reduce):** When a node i in state $s_i = A$ receives a message informing the transition of node j from state $s_j = L$ to $s_j = B$, its effective degree e_i changes. Then, node i has to send its updated effective degree. While the exact number of messages sent informing the changes in effective degree depends on the topology of the network, we can compute its upper bound to be $M_5 = Nd$, where d is the average degree of the nodes in the network. The reasoning behind this result is as follows.

  Consider a node j which is transitioning from $s_j = L$ to $s_j = I$. This will lead to a change in effective degree of all its neighbors. Out of these neighbors, only the nodes in active state send a message informing the change in effective degree. Therefore the number of update messages sent is equal to the number of edges between the node j and its active neighbors. It also follows that no update message would be sent by the node j after it has transitioned to the inactive state. Therefore, for the purpose of counting the number of update messages, we must iteratively 'remove' the nodes which have transitioned to the inactive state along with all their edges. The total number of edges removed thus would give the upper bound of the number of update messages sent. Since the maximum number of edges in the network is Nd, therefore the maximum number of update messages is $M_5 = Nd$.

- **Broadcasting messages from the residue nodes (message type h = broadcast):** In the final stage of the algorithm, each node in the residue state creates a broadcast message which is then broadcast

throughout the network. If there are r nodes reaching the residue state, the number of messages sent in this stage is $M_6 = Nr$.

The upper bound of the total number of messages sent in the entire duration of the algorithm is, therefore,

$$M = \sum_{k=1}^{6} M_k = 4N - Nx + Nd + N^2 x \tag{45}$$

where,

$$x = \frac{r}{N} \tag{46}$$

is the fraction of residue nodes in the network.

In the All-2-All broadcast method for node counting, each of the N nodes sends its id to all the N nodes of the network. Therefore, the total number of messages sent in the algorithm is $M_0 = N^2$.

Comparing the two algorithms in terms of the communication costs, we can say that the proposed algorithm is better if

$$4N - Nx + Nd + N^2 x < N^2$$

or equivalently if

$$d < N(1-x) + x - 4. \tag{47}$$

In other words, the proposed algorithm is better than the All-2-All broadcast method if the average degree of the nodes is less than the threshold on the right-hand side of Eq. 47. This threshold depends on the size of the network N and the fraction of residue nodes x. Since $0 < x \leq 1$, we now analyze Eq. 47 in the limiting cases.

If $x = 1$, all the nodes of the network have gone through the residue state. This occurs in the special case when all nodes of the network have the same degree (in other words, we have a regular network). In such a limiting case, Eq. 47 reduces to $d < -3$, which is impossible. Therefore, in regular networks of any size the direct broadcast method is better than the proposed algorithm in terms of communication costs. On the other hand, if $x \to 0$, Eq. 47 becomes $d < N - 4$. Since in a typical network, the average degree of a node is much less than the number of nodes, we can say that the proposed method is better for almost any network where the fraction of residues is close to zero.

When applied to random graphs of aforementioned types in simulations, we observe that the fraction of residue nodes x comes out to be close to zero for sufficiently large networks (see Figure 2 bottom panel). For instance, Barabási Albert, Erdös Rényi and Watts-Strogatz networks of size 10,000 have the fraction of residue nodes x below 0.02. This implies that the threshold average degree d for which the proposed algorithm outperforms the All-2-All broadcast method is $d > 9796$. Even for a Random Geometric network, where $x \approx 0.07$, the threshold is approximately $d \approx 9300$. Since the approximate average degrees for the networks is approximately $d \approx 20$ to 30, which is significantly less than the indicated thresholds, we can conclude that the proposed algorithm has significantly lower communication costs than the All-2-All broadcast method. Moreover, since Figure 2 indicates that x remains constant, or decreases, as the network size increases, we expect that the proposed algorithm would be even more efficient for larger networks.

0.1 Communication Cost of the ST Algorithm

The number of messages sent across the network is at least $2N^2$. More specifically, each node sends at least two messages for each query it receives: one to establish the hierarchy during the tree construction phase and one to send towards the root the count of the nodes. Since each node computes the ST algorithm independently, each node receives N queries, and therefore it sends at least $2N$ messages. We can thus derive that the total number of messages sent across the network is at least $2N^2$. The asymptotic worst-case complexity in terms of communication for the ST algorithm is therefore $\mathcal{O}(N^2)$.

0.2 Memory Cost

Throughout the execution of the algorithm, each node keeps track of a number of internal variables. The state variable s_i can take one of four different values and therefore, has memory requirements independent of the network properties. Since these memory requirements are relatively small, we ignore them in the further analysis.

The variables c_i, n_i and e_i can be numbers up to and including N. Hence, the memory requirement for each of them is proportional to $\log N$. Similarly, the memory requirement for any message variable $m_{i,h}$ can scale as $2 \log N$

in the worst cast scenario since $m_{i,h}$ is either a single number or a tuple containing 2 numbers which are all bounded above by N.

However, the variables $\mathcal{N}_i$, $\mathcal{E}_i$ and $\mathcal{R}_i$ are sets whose memory requirements are much larger than the previously mentioned single valued variables. The number of elements in $\mathcal{N}_i$ is the degree of the node d_i. Since each of the elements is the index of a node, the memory required to store each element is proportional to $\log N$. Therefore, the memory requirement for the set is, $M(\mathcal{N}_i) \sim 2d_i \log N$. The maximum memory requirement for $\mathcal{E}_i$ is exactly two times that of $\mathcal{N}_i$ because the initial number of elements in $\mathcal{E}_i$ and each element is a tuple of two numbers, each of which requires memory proportional to $\log N$. Therefore, $M(\mathcal{E}_i) \sim d_i \log N$. Note that, since $\mathcal{N}_i$ is used only to construct the elements of $\mathcal{E}_i$, the memory used for storing $\mathcal{N}_i$ can simply be expanded to store $\mathcal{E}_i$. The elements of $\mathcal{R}_i$ are also tuples whose memory requirements are similar to those in $\mathcal{E}_i$. However, the number of such tuples in $\mathcal{R}_i$ is equal to the number of residues r in the network, Hence, $M(\mathcal{R}_i) \sim r \log N$. Therefore, the maximum memory requirement for each node i scales as,

$$M_i \sim (2d_i + r + 5) \log N \tag{48}$$

for large N. In comparison, if the number of nodes is computed using the All-2-All broadcast method, each node requires memory that scales as $N \log N$ as it needs to keep track of indices of every other node of the network. In the worst case scenario, the degree of each node of the network can be $d_i = N - 1$ for all nodes which also implies that all nodes become residue nodes, yielding $r = N$. In such a case, the memory requirement for the proposed algorithm scales as $3N \log N$ for large N which is clearly worse than the All-2-All broadcast method. However, our numerical simulations involving much general classes of random networks show that r is at least one order of magnitude smaller than N (see Figure 2 bottom panel) and the degree of each node is approximately the same (due to the parameters chosen in Table 3) for sufficiently large networks. Therefore, we can say that the proposed algorithm is better than the All-2-All broadcast method for sufficiently large networks.

0.3 Memory Cost of the ST Algorithm

For each query received, each node has to keep track of the id of the querying node and of the id of its parent for the corresponding query. Because there

Table 1: Asymptotic worst-case complexity for the AnB, the All-2-All, and the Single Tree (ST) algorithms in terms of time, communication, and memory. In the worst-case the three algorithms are comparable in every aspect (except for ST's memory) however, the precise memory and computational costs equations of Table 2 in the main text show that the AnB algorithm is more efficient in most cases.

Algorithm	Time	Communication	Memory
AnB	$\mathcal{O}(N)$	$\mathcal{O}(N^2)$	$\mathcal{O}(N\log(N))$
All-2-All	$\mathcal{O}(N)$	$\mathcal{O}(N^2)$	$\mathcal{O}(N\log(N))$
ST	$\mathcal{O}(N)$	$\mathcal{O}(N^2)$	$\mathcal{O}(N^2)$

Table 2: Comparison of algorithms for Ring and Complete Networks.

Mode	Ring Network		Complete Network	
	Communication	Memory	Communication	Memory
AnB	$N(2N+3)$	$(3N+5)\log N$	$N(N+5)$	$(N+9)\log N$
All-2-All	N^2	$N\log N$	N^2	$N\log N$
ST	$2N^2$	$2N\log N + N^2$	$2N^2$	$2N(\log N + 1)$

are N queries and storing an id requires at least $\log(N)$ bits, the memory required by each node to keep track of its parents is at least $2N\log(N)$. Additionally, each node has to ensure that it receives messages from all its neighbors for each query. This requires an additional d_iN bits, where d_i is the degree the node i. Therefore, the total memory required by the generic node i is $2N\log(N) + d_iN$.

0.4 Performance on Ring and Complete Networks.

In the paper, we show that, for a general network, the AnB algorithm performs better than the All-2-All and ST algorithms in terms of the memory and communications costs. However, the AnB algorithm would be outperformed by the All-2-All and ST algorithms if the network topology is such that a large number of Residue nodes are created. The completely connected and the ring networks are such classes of networks. Since, the degree of each node is same in these networks, all nodes go through the residue state. In such cases, we have (see Table 2),

- For Complete Network: $d_i = r_i = N \;\; \forall i$. Therefore, the communication and memory costs from Table 2,
- For Ring Network: $d_i = 2, r_i = N \;\; \forall i$. Therefore, the communication and memory costs from Table 2,

Hence, the AnB algorithm is outperformed by All-2-All algorithm in terms of communication and memory costs for complete and ring networks. The AnB algorithm is outperformed by ST algorithm in terms of communication costs for these networks. However, even for these networks, the AnB algorithm is still better than ST algorithm in terms of memory costs.
